# State of the Art in Pancreatic Surgery: Some Unanswered Questions

**DOI:** 10.3390/jcm11102821

**Published:** 2022-05-17

**Authors:** Teresa Perra, Alberto Porcu

**Affiliations:** Department of Medical, Surgical and Experimental Sciences, University of Sassari, Viale San Pietro 43, 07100 Sassari, Italy; alberto@uniss.it

Pancreatic surgery is one of the most technically challenging types of surgery, and many questions remain unanswered; therefore, an overview of the global advancement in surgical research and clinical practice is fundamental in this field.

As there is a global consensus that pancreatic surgery is a major clinical procedure, it requires adequate expertise and experience [1,2,3].

Several surgical techniques and different anastomoses for the reconstruction of the digestive system have been performed over the years. Generating reports within a uniform, standardized framework plays an important role in this field of research. A classification system was proposed by the International Study Group of Pancreatic Surgery (ISGPS) in 2010. They underline the importance to report factors related to the pancreatic remnant (e.g., pancreatic duct size, length of mobilization, gland texture), and the pancreatoenteric anastomosis, such as the use of pancreatojejunostomy/pancreatogastrostomy, duct-to-mucosa anastomosis, invagination (dunking) of the remnant into the jejunum or stomach, and the use of a stent across the anastomosis [4].

The available data do not show any definitive advantages for one specific type of gastrointestinal reconstruction technique after pancreatoduodenectomy, compared with other techniques [5,6,7].

Pancreatic surgery has been adapted to treat different pancreatic diseases (e.g., cancer, acute or chronic pancreatitis, cysts).

Pancreas transplantation is a curative treatment for type 1 diabetes mellitus. Current evidence shows that it has the highest complication rate among organ transplants. There is still little evidence regarding the best option for revascularization of the transplanted organ, and therefore, randomized trials are important in this field [8].

Various factors influence cancer recurrence, morbidity, and mortality after pancreatic surgery. Some of these are known, while others have been poorly studied. The evaluation of nutritional status has gained increasing attention, especially in recent years, indicating that it should be part of routine preoperative assessment [5].

The most common and clinically relevant complications are related to pancreaticojejunal anastomosis. In addition, the risk of postoperative pancreatic fistula [9], bile leak, delayed gastric emptying, postpancreatectomy hemorrhage, and chyle leak deserve consideration.

Resectability criteria, as well as absolute and relative contraindications to pancreatic surgery, are re-evaluated in light of new scientific evidence.

Advanced techniques of surgical resection and vessel reconstruction can allow curative-intent surgery in borderline resectable and locally advanced pancreatic ductal adenocarcinoma (PDAC).

Elaborated techniques of surgical resection and vessel reconstruction have been performed to enhance the completeness of resection and reduce the risk of local recurrences, which include artery-first and uncinate-first approaches, dissection of the anatomical triangle between the coeliac and superior mesenteric arteries and the portomesenteric vein, and radical antegrade modular pancreatosplenectomy. Advanced techniques for resection and reconstruction of the mesenteric–portal vein axis and a venous bypass graft-first approach can allow resection of PDAC with venous involvement. Arterial involvement does not preclude surgical resection but may become surgically manageable following neoadjuvant treatment [10].

A consensus statement by ISGPS establishes that current evidence supports operative exploration and resection in the case of involvement of the mesentericoportal venous axis. It also supports exploration to confirm the imaging-based findings in the case of suspicion of arterial involvement [11].

Although pancreatoduodenectomy (PD) with the resection of portal–mesenteric vein axis has significantly increased the resection rate in patients with pancreatic head adenocarcinoma, it seems to have lower survival outcomes and higher 30-day mortality, compared with standard PD, whereas postoperative morbidity rates are similar [12].

More investigations are needed on the effects of surgical resection and vessel reconstruction on overall survival [10].

Furthermore, the choice between neoadjuvant therapy and upfront surgery is debated, in particular in the case of vascular reconstruction.

Emergency pancreatic surgery is rarely reported in the medical literature. However, emergency pancreatoduodenectomy can represent a life-saving procedure for complex pancreaticoduodenal injuries in selected patients.

There are still many unanswered questions, and further studies are needed to better manage and treat surgical patients with pancreatic disease.

Sharing researchers’ experiences and discussing surgical approaches are fundamental to providing an overview of the global advancement of surgical research and clinical practice in the field of pancreatic surgery.
